# Homozygous UNC13A Variant in an Infant With Congenital Encephalopathy and Severe Neuromuscular Phenotype: A Case Report With Detailed Central Nervous System Neuropathologic Findings

**DOI:** 10.7759/cureus.30774

**Published:** 2022-10-27

**Authors:** Jordyn R Mullins, Kathryn McFadden, Nicole Snow, Angelica Oviedo

**Affiliations:** 1 Pathology and Laboratory Medicine, Burrell College of Osteopathic Medicine, Las Cruces, USA; 2 Pathology and Laboratory Medicine, IWK Health Centre and Dalhousie Medical School, Halifax, CAN; 3 Medical Genetics, IWK Health Centre and Dalhousie Medical School, Halifax, CAN

**Keywords:** unc13a, cerebellar purkinje cell gliosis, axonal spheroids, sub-cortical u-fibers, central nervous system development, neuromuscular junction, synapse

## Abstract

Uncoordinated 13 (*UNC13A*) affects movement in *Caenorhabditis elegans* (*C. elegans*). It is responsible for docking, priming, and stabilizing synaptic vesicle fusion complexes in the neuronal synapse and neuromuscular junction (NMJ). It also plays an important role in central nervous system development. We report the detailed clinical history and central nervous system neuropathologic findings in an infantile case with homozygous *UNC13A* loss of function variant, in order to advance the understanding of this critically important synaptic vesicle protein. This is the first detailed central nervous system neuropathologic report of this rare case of homozygous *UNC13A *loss.

## Introduction

Uncoordinated 13 (*UNC13A*) was discovered in 1974, during screening for genetic variants that affect behavior in *Caenorhabditis elegans* (*C. elegans*) [1]. *UNC13A* plays a role in the docking and fusion of synaptic vesicles at the central neuronal and neuromuscular synaptic junctions through interaction with soluble N-ethylmaleimide sensitive attachment protein receptor (SNARE) [2-3]. *UNC13A* is involved in the rapid release of neurotransmitters at both the cholinergic neuromuscular synapse and central gamma-aminobutyric acid (GABA) and glutamatergic synapses [3-5]. *UNC13A* is a member of the UNC13 protein family, which is involved in controlling presynaptic vesicle fusion [6]. It is also involved in synaptic plasticity mechanisms [6].

In mammals, three homologous genes, on three different chromosomes, are referred to as *UNC13* due to their striking similarity in function. *UNC13A, UNC13B, and UNC13C *are located on chromosomes 19, 9 and 15, respectively [7-9]. All three have been identified throughout the central nervous system (CNS). In addition, *UNC13A* also plays a role at the neuromuscular junction (NMJ) [3]. The molecular action of *UNC13A* has been extensively studied using knock-out mouse models [5-6]. Double knockout (DKO) of *UNC13A/B* and triple knockout (TKO) of *UNC13A/B/C* in the mouse caused complete paralysis and death. The DKO of *UNC13-B/C* resulted in viability and fertility. Therefore, *UNC13A* plays a critical role in the development and function of the CNS [3]. In addition, a reduction of *UNC13A* at the presynaptic terminal is correlated with a decrease in synaptic strength, regulation, and plasticity [6].

In addition to the role that *UNC13A* plays in normal nervous system and brain development, loss of function likely plays a role in neurodegenerative disease. *UNC13A *has been shown to play a role in motor neuron survival in a *C. elegans* model of amyotrophic lateral sclerosis (ALS) [5]. In addition, certain genetic polymorphisms in *UNC13A* cause increased risk of both sporadic ALS and sporadic frontotemporal degeneration (FTD) [10]. Whole genome association studies have shown that certain *UNC13A* single nucleotide polymorphisms (SNPs) are a significant risk locus in the development of ALS and FTD [11].

Here, we report the first detailed gross and microscopic central nervous system neuropathologic findings from an autopsy of an infant with congenital encephalopathy and homozygous loss-of-function variant in *UNC13A*. We also report the electron microscopy findings from a skin biopsy done during life. These findings along with the severe clinical phenotype help elucidate the role of *UNC13A* in brain function, development, and neurodegeneration.

## Case presentation

A G1P0 mother at 40 weeks gestation delivered a female neonate by cesarean section for decreased fetal movements and abnormal fetal heart rate. Prenatal history was otherwise unremarkable. The infant was born at a community hospital in good condition (Apgar score of 9 at 1 and 5 min). She was admitted to the post-partum unit with her mother but was transferred to a Neonatal Intensive Care Unit in a tertiary care center on day 3 of life for persistent hypotonia and poor feeding. She had mildly dysmorphic features consisting of bitemporal narrowing, a short forehead, short palpebral fissures, a short upturned nose with slightly underdeveloped central columella (nares slightly lower than columella), and a short neck. Her clinical course included severe neurological findings with a history of alternating hypertonia and hypotonia, decreased consciousness, limited activity, weak suck and gag reflexes, extreme generalized muscle weakness, and irritability. Additional findings over the course of her life included severe kyphoscoliosis and diaphragmatic and umbilical hernias. Due to poor feeding, she was fed through a gastrojejunostomy tube. Ophthalmologic examination showed small optic nerves accompanied by prominent right iris vasculature. The mother had a history of Wolff-Parkinson-White Syndrome along with a duplicated ureter. The father was normal and healthy. There were no other familial genetic disorders. The parents are distant cousins. 

Magnetic resonance imaging of the brain showed non-occlusive venous sinus thrombosis that was considered not clinically significant but otherwise was normal. Electroencephalogram (EEG) revealed a burst suppression pattern, which correlated with infantile spasms. Laboratory studies showed normal ammonia, serum amino acids, and urine organic acids. No muscle biopsy was done during life.

Her condition deteriorated and she died at the age of 8 months. The parents gave consent for an unrestricted autopsy. The lungs showed bronchopneumonia, which was considered the cause of death in the setting of severe neurological disease. The liver showed mild vacuolization on hematoxylin and eosin stains, interpreted as end-stage changes. Peri-venular hepatocytes showed periodic acid-Schiff (PAS) and PAS-diastase faintly positive, small, globular cytoplasmic inclusions (one to three inclusions per hepatocyte). Although the role of UNC13A in hepatocytes is not known, these hepatocyte inclusions (not shown) are considered part of the UNC13A loss of function phenotype. Clinically, this patient had no known liver abnormalities. Skeletal muscle showed significant artifacts and could not be interpreted. Systemic organ findings were otherwise unremarkable. 

**Figure 1:**
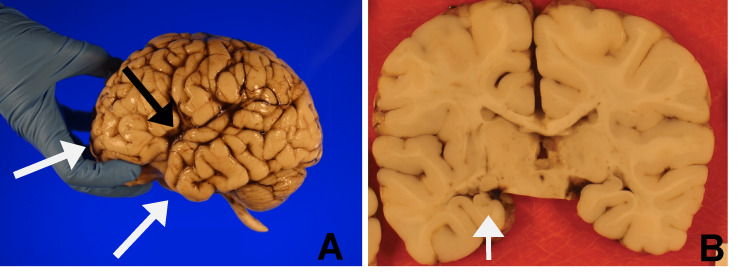
Gross brain. A: Open Sylvian fissure (black arrow) with blunted bilateral frontal lobes and left temporal lobe (white arrows). B: Left hippocampus with vertical orientation (arrow) on coronal section of formalin-fixed brain.

On autopsy, the fresh brain weighed 897 g, which was considered within normal limits for age. External examination (Figure 1A) of the formalin-fixed brain showed open Sylvian fissures (0.6 cm bilaterally). The bilateral frontal and left temporal lobes appeared blunted. Coronal sections of the formalin-fixed brain showed a vertical orientation of the left hippocampus (Figure 1B) and bilaterally absent lines of Gennari in the occipital lobes (not shown). A microscopic examination of the hippocampus confirmed the abnormal orientation but was otherwise unremarkable (not shown). 

**Figure 2:**
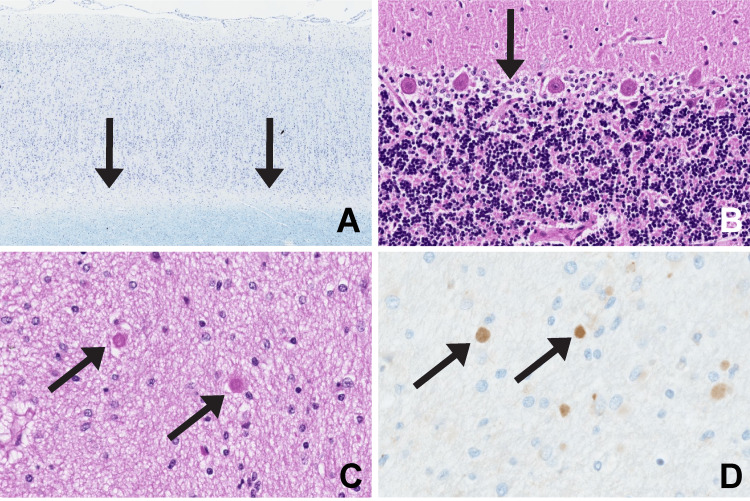
CNS neuropathology. A: Frontal cortex shows absent sub-cortical U-fibers (arrows) with LFB. B: Cerebellum shows gliosis of the Purkinje cell layer without neuronal loss (arrow) with HE. C: Septum pellucidum shows axonal spheroids (arrows) with HE. D: Axonal spheroids are positive for synuclein by immunohistochemistry (arrows). HE: hematoxylin and eosin stain; CNS, central nervous system; LFB, luxol fast blue stain

Light microscopy (Figure 2) showed absent subcortical U-fibers on both Luxol Fast Blue (LFB) and Bielschowsky stains in all cortical areas examined. The septum pellucidum showed round bodies consistent with axonal spheroids, which were synuclein positive on immunohistochemistry (Alpha-synuclein Monoclonal Antibody, ThermoFisher Scientific, Waltham, MA). The cerebellum showed moderate gliosis of the Purkinje cell layer without Purkinje cell loss. The spinal cord sections showed widened anterior fissure and expanded peri-vascular spaces, interpreted as moderate atrophy due to tissue loss, while the optic nerves appeared small (not shown). The caudate, putamen, thalamus, and brainstem sections were grossly and microscopically unremarkable. Immunohistochemistry for TDP-43 on the hippocampus and cerebellum showed no nuclear inclusions and no cytoplasmic inclusions (not shown).

**Figure 3:**
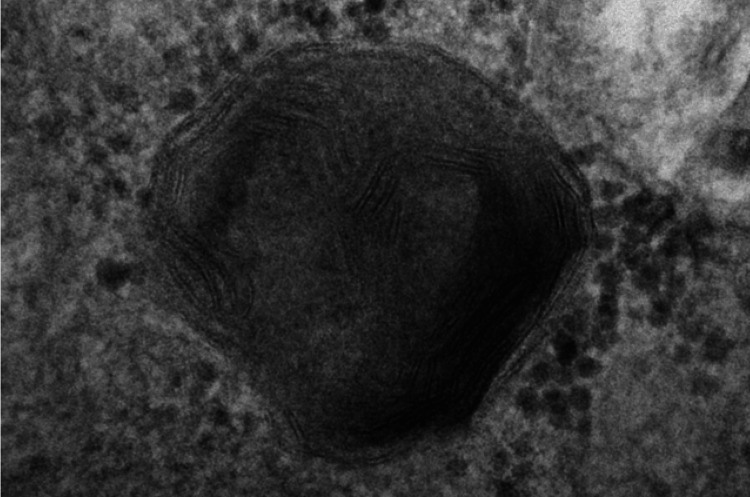
Electron microscopy skin. Fingerprint body present on skin biopsy

Skin biopsy done during life showed fingerprint-like and lamellar inclusions in several different cell types by electron microscopy (Figure 3). 

Exome sequencing done during life revealed a homozygous UNC13A nonsense variant, c.1188delC p.(Asp397Thrfs*107) that created a frame shift and resulted in a stop codon 106 positions downstream of codon 397. An epileptic encephalopathy panel was negative for any relevant variants.

## Discussion

Our case study highlights the clinical history and detailed CNS neuropathologic findings in this infant with *UNC13A* loss of function variant. Given its critical role in brain development and synaptic function, loss of *UNC13A* would be expected to result in neuropathological abnormalities; this is seen in our patient as blunted frontal and temporal lobes, open Sylvian fissures, absence of sub-cortical U-fibers, axonal spheroids in the septum pellucidum, cerebellar Purkinje cell gliosis, spinal cord atrophy, and small optic nerves. The skin also demonstrated fingerprint bodies on electron microscopy.

Engel and colleagues (2016) described the first identified human case of *UNC13A* loss of function variant, which had a similar clinical history to our case including hypotonia, poor feeding, gastroenterostomy tube, kyphoscoliosis, frontotemporal narrowing, eye abnormalities, EEG abnormalities, spinal cord atrophy, and fetal demise due to aspiration pneumonia [4]. The only major phenotypic difference was the first case was microcephalic (by head circumference) while our case had normal brain weight at autopsy. Also, the first case was born at 32 weeks gestation while our case was born at term. The first described patient had a homozygous nonsense *UNC13A* variant that caused retention of only 101 of 1703 amino acid residues of the corresponding protein. Engel and colleagues described extremely detailed skeletal muscle physiology including in-vitro muscle studies. Electromyogram (EMG) studies showed low-amplitude compound muscle action potentials (CMAPs) with a decrement of the fourth compared with the first evoked CMAP and findings consistent with a pre-synaptic defect. That infant had a protracted clinical course and died at the age of 4 years. No autopsy was done.

Since the final development and fine-tuning of the central nervous system is dependent on synaptic transmission [3], it is expected that *UNC13A* loss would cause abnormalities in the affected tracts. The abnormally formed frontotemporal lobes, hippocampus, and loss of U-fibers in our case are likely the result of the pathways affected by the *UNC13A* loss. A mouse model of *UNC13A* loss showed markedly abnormal neuronal electrophysiology in the hippocampus [12], which we correlate with the abnormally formed hippocampus in our infant. The rodent hippocampus has uncomplicated development relative to the human hippocampus, so is less likely to show morphologic abnormalities in the setting of neuronal dysfunction. In addition, it is likely that there are compensatory mechanisms that allow for some brain development, but are not adequate for normal development and function at later stages.

Our case showed synuclein-positive axonal spheroids in the septum pellucidum. The cortex, thalamus, substantia nigra, and cerebellum were all negative for synuclein; no neuronal inclusions were seen. The synuclein positivity confirms the interpretation of the septum pellucidum round bodies as axonal spheroids [13], but no additional interpretation is possible in the setting of absent synuclein-positive neuronal inclusions. The findings of neuroaxonal dystrophy were not seen. We interpret the septum pellucidum axonal spheroids as part of the *UNC13A* phenotype. 

Our case showed gliosis of the cerebellar Purkinje cell layer without neuronal loss. Cerebellar abnormalities have been described in a mouse model of *UNC13C* loss. *UNC13C* is another member of the synaptic complex expressed largely in the cerebellum in the rat model. Studies in the mouse have shown that *UNC13C* is involved in the development and communication of cerebellar Purkinje cells [14]. We consider the cerebellar Purkinje cell gliosis secondary to the *UNC13A* loss in our infant since UNC13A and UNC13C are both parts of the same synaptic complex. We interpret this finding as gliosis which is reactive to cerebellar neuronal synaptic abnormalities. 

The first identified human case of *UNC13A* loss had a very similar clinical history to our case. That case report described the muscle pathology in exquisite detail [4]. Unfortunately, skeletal muscle studies were not done on our patient. Our case completes the neuropathologic phenotype with detailed CNS neuropathologic findings in the brain. In addition, the remarkable parallels to the animal models of *UNC13A* loss validate those models for the study of human *UNC13A* loss. The numerous abnormal findings in these patients will allow for clinical identification of patients suspected of having congenital abnormalities of the pre-synaptic neuronal and NMJs.

## Conclusions

We present this case of homozygous *UNC13A* loss of function variant to show the associated detailed CNS neuropathologic phenotype. Gross abnormalities, including open Sylvian fissures, blunted frontal and temporal lobes and a malformed hippocampus, were seen. Numerous microscopic abnormalities including axonal spheroids in the septum pellucidum, loss of sub-cortical U-fibers, and cerebellar Purkinje cell layer gliosis were seen. It is important to describe this rare syndrome so that healthcare providers can recognize this and related entities during life. Previous animal models have significant overlap with our case and validate those models for the study of *UNC13A* and related proteins. Further study of *UNC13A* loss is important so that potential therapies may be found.
